# Data-Efficient Language Model for Assessing Pulmonary Embolism Diagnostic Certainty From Radiology Reports: Model Development and Validation Study

**DOI:** 10.2196/79972

**Published:** 2026-04-28

**Authors:** Feifan Liu, Ruofan Hu, Donghyuk Kim, Hao Lo, Yevgeniy Kharonov, Ansh Johri, Ben S Gerber, Elke Rundensteiner, Lauren M Westafer, Jeroan Allison, Catarina Kiefe, Alexander Bankier, Max P Rosen

**Affiliations:** 1 Chan Medical School, University of Massachusetts, 55 Lake Avenue North, Worcester, MA, 01655, United States, 1 (508) 856-8924, 1 (508)-856-8993; 2 Data Science, Worcester Polytechnic Institute, Worcester, MA, United States; 3 Department of Radiology, University of Miami Miller School of Medicine, Miami, FL, United States; 4 Chan Medical School, University of Massachusetts, Baystate, MA, United States

**Keywords:** pulmonary embolism, radiology reports, natural language processing, machine learning, diagnostic certainty

## Abstract

**Background:**

Computed tomography pulmonary angiography (CTPA) is the standard imaging modality for diagnosing pulmonary embolism (PE), but diagnostic uncertainty is common due to technical limitations and vague language, leading to inconsistent interpretation and clinician frustration.

**Objective:**

This study develops a prompt-free, data-efficient method for assessing diagnostic certainty of PE in CTPA reports using small pretrained language models.

**Methods:**

This study examined 173 consecutive CTPA reports from UMass Memorial Health, each annotated by 3 radiologists for PE diagnostic certainty. We developed PECertainty, a lightweight, prompt-free model, and compared it with advanced large language model (LLM)–based methods under limited supervision settings. Baselines included prompt-free methods (support vector machine, random forest, and RoBERTa [Robustly Optimized Bidirectional Encoder Representations From Transformers Pretraining Approach]) and prompt-dependent methods (LLM fine-tuning, in-context learning, and ADAPET [A Densely-Supervised Approach to Pattern Exploiting Training]; UNC Chapel Hill) with open-source Gemma3-4B (Google DeepMind) and Llama3.2-3B (Meta), and the proprietary GPT-3.5 (OpenAI). Sensitivity analyses evaluated performance with 1 to 10 training examples per category for the top performer. Model performance was evaluated against radiologist annotations. External validation on 420 CTPA reports from the Baystate Medical Center, with validation limited to distinguishing certain from uncertain reports. Interpretability of the top-performing models (PECertainty and GPT-3.5) was evaluated using integrated gradients and prompt-based explanations reviewed by radiologists.

**Results:**

Among prompt-dependent methods, GPT-3.5 fine-tuning (*F*_1_-score 0.86; 95% CI 0.71‐1.0) and in-context learning (*F*_1_-score 0.87; 95% CI 0.71‐1.0) performed best, and the performance of in-context learning consistently outperformed 0-shot learning for Gemma3-4B (*F*_1_-score 0.63, 95% CI 0.56‐0.79 vs *F*_1_-score 0.45; 95% CI 0.29‐0.56) and Llama3.2-3B (*F*_1_-score 0.54; 95% CI 0.41‐0.71 vs *F*_1_-score 0.43, 95% CI 0.28‐0.62). PECertainty demonstrated numerically better or equivalent performance compared with both the top-performing prompt-dependent methods and all prompt-free baselines. Compared with fine-tuned ClinicalBERT (Bidirectional Encoder Representations From Transformers Pretrained on Clinical Text), PECertainty achieved statistically significant improvements across all metrics (paired bootstrap significance test, *P*<.05). RoBERTa (Robustly Optimized Bidirectional Encoder Representations From Transformers Pretraining Approach) fine-tuning lagged (*F*_1_-score 0.52; 95% CI 0.35‐0.71), and simple models such as support vector machine underperformed. In few-shot settings (10 examples/category), PECertainty (*F*_1_-score 0.80; 95% CI 0.59‐0.94) outperformed both GPT-3.5 fine-tuning (*F*_1_-score 0.74; 95% CI 0.58‐0.88) and in-context learning (*F*_1_-score 0.65; 95% CI 0.47‐0.83). External validation on the Baystate dataset showed good generalization for distinguishing certain from uncertain cases (*F*_1_-score 0.77; 95% CI 0.70‐0.83). Despite its strong performance, PECertainty was rated as less interpretable than fine-tuned GPT-3.5 by radiologists (*t* test, *P*<.05).

**Conclusions:**

PECertainty enables accurate and data-efficient assessment of diagnostic certainty from free-text CTPA reports in low-resource settings. As an open-source, lightweight alternative to proprietary LLMs, it may support more precise communication between radiologists and referring physicians, with interpretability identified as a key direction for improvement.

## Introduction

Diagnostic certainty in evaluating pulmonary embolism (PE) is critical, as both missed diagnoses and overdiagnosis can result in serious patient harm, including delayed life-saving treatment or unnecessary anticoagulation with associated bleeding risks. In the emergency department (ED), prompt evaluation of suspected PE is essential for risk assessment and treatment. Computed tomography pulmonary angiography (CTPA) is widely used as the diagnostic standard due to its accessibility and high sensitivity. However, CTPA has limitations, including variability in contrast timing and respiratory motion artifacts. Thus, diagnostic uncertainty in CTPA reports is common and usually conveyed in vague language that can be inconsistently interpreted.

Referring clinicians often express frustrations with ambiguous linguistic hedging, emphasizing the need for clearer and more consistent communication from radiologists as a top priority for improving care quality [1-3]. For example, a reporting statement “No evidence of pulmonary embolism to the lobar level. No airspace or interstitial disease. Mild left axillary lymphadenopathy” appears reassuring at first glance, yet the hedge phrase “to the lobar level” introduces a hedge that indicates reduced diagnostic confidence, as it suggests that while no PE is observed at the lobar level, emboli in the more distal segmental or subsegmental arteries cannot be definitively ruled out. Such subtle expressions of uncertainty can have significant effects on clinical practice. In the high-pressure ED setting, unclear language on diagnostic reporting can give false reassurance or result in overly cautious clinical decisions. Both situations can harm patient outcomes. Thus, clear communication between radiologists and clinicians is essential to ensure accurate diagnosis, improve patient care, reduce mortality, and prevent unnecessary anticoagulation [4,5], especially in the ED setting where clinicians have to make quick and accurate treatment decisions.

Previous efforts to improve report clarity have focused on structured reporting systems that standardize phrases such as “consistent with (>90% certainty)” and “possibly (≈50% certainty)” to improve diagnostic reporting [6]. However, these approaches often focus narrowly on lexicon standardization, overlooking important contextual information such as “rambling” differential diagnoses, using long and vague sentences, and indicating technical limitations. The predefined certainty categories are typically broad and may lack meaningful interpretability for treating clinicians. Although structured reporting is promising, there are recognized limitations. These include interference with radiologist image interpretation, reduced descriptive power by limiting expressive flexibility, added burden of learning and using preferred terminologies, and required availability of lexicons and templates across imaging disciplines (not generalizable) [7,8]. These templates are often institution-specific, further limiting their generalizability across health care settings.

Studies found that a structured report accompanied by a radiologist’s comment is the most preferred style by referring physicians [9]. The use of speech-to-text dictation remains desirable, where radiologists use natural language as though speaking to a human transcriptionist [7]. Successful integration of dictated comments and structured reporting can help bridge the preference gaps in report format and style between radiologists and referring physicians [10] and address issues related to adopting new technological advances [11]. For this integration to be successful, a standardized and calibrated context-aware uncertainty measurement is needed for improved diagnostic uncertainty communication in radiology reporting.

Advances in natural language processing (NLP) with fine-tuning pretrained language models (PLMs) have been successfully applied to different tasks in radiology, including clinical information extraction [12], report generation [13], abnormal sentence classification [14], report coding [15], and diagnostic certainty assessment [16]. However, few studies explored advanced NLP for PE diagnostic certainty assessment, especially when annotated data is limited. Chapman et al [17] developed a rule-based system only to detect uncertainty in PE diagnostic reports (certain vs uncertain), achieving 93% accuracy. However, their approach relied on manually curated rules, which are labor-intensive, nonscalable, and unable to capture probabilistic gradations or longer contextual dependencies. Additionally, the system could not effectively model the interaction between diagnostic findings (positive or negative) and certainty levels (certain or uncertain), limiting its clinical utility.

To address the aforementioned limitations, we developed a lightweight, open-source model, PECertainty, to assess the level of diagnostic certainty expressed in radiology reports for PE. Our goal was to build a solution that performs well even with limited labeled data and does not rely on manually crafted prompts or access to large proprietary models, such as GPT-3 [18]. To this end, we adopted the prompt-free and data-efficient SetFit framework [19] to fine-tune smaller PLMs. We evaluated the performance of PECertainty against a range of strong baselines, including prompt-free methods (SVM [support vector machine], random forest, and RoBERTa) and prompt-dependent approaches, such as large language model (LLM) fine-tuning, in-context learning, and ADAPET [A Densely-Supervised Approach to Pattern Exploiting Training]. The LLMs evaluated include open-source models (Gemma3-4B and Llama3.2-3B) as well as the proprietary GPT-3.5. We further assess PECertainty under varying training data sizes to evaluate its robustness in low-resource settings. To examine its generalization capability, we test PECertainty on an external dataset. Finally, we compare the interpretability of PECertainty with that of competing models based on radiologist evaluations.

## Methods

### Ethical Considerations

This study was approved by the Institutional Review Board at the University of Massachusetts Chan Medical School (IRB# H00022301). This study involved a retrospective analysis of existing, deidentified clinical text data, and the requirement for informed consent was waived in accordance with applicable federal regulations (45 CFR 46.104[d](4)) [20]. No protected health information or personally identifiable information was accessed or used in this study. All data were deidentified before analysis, and appropriate safeguards were implemented to maintain participant privacy and data confidentiality, including secure data storage and restricted access protocols. Participants received no compensation.

### Study Design and Datasets

**Figure 1.**
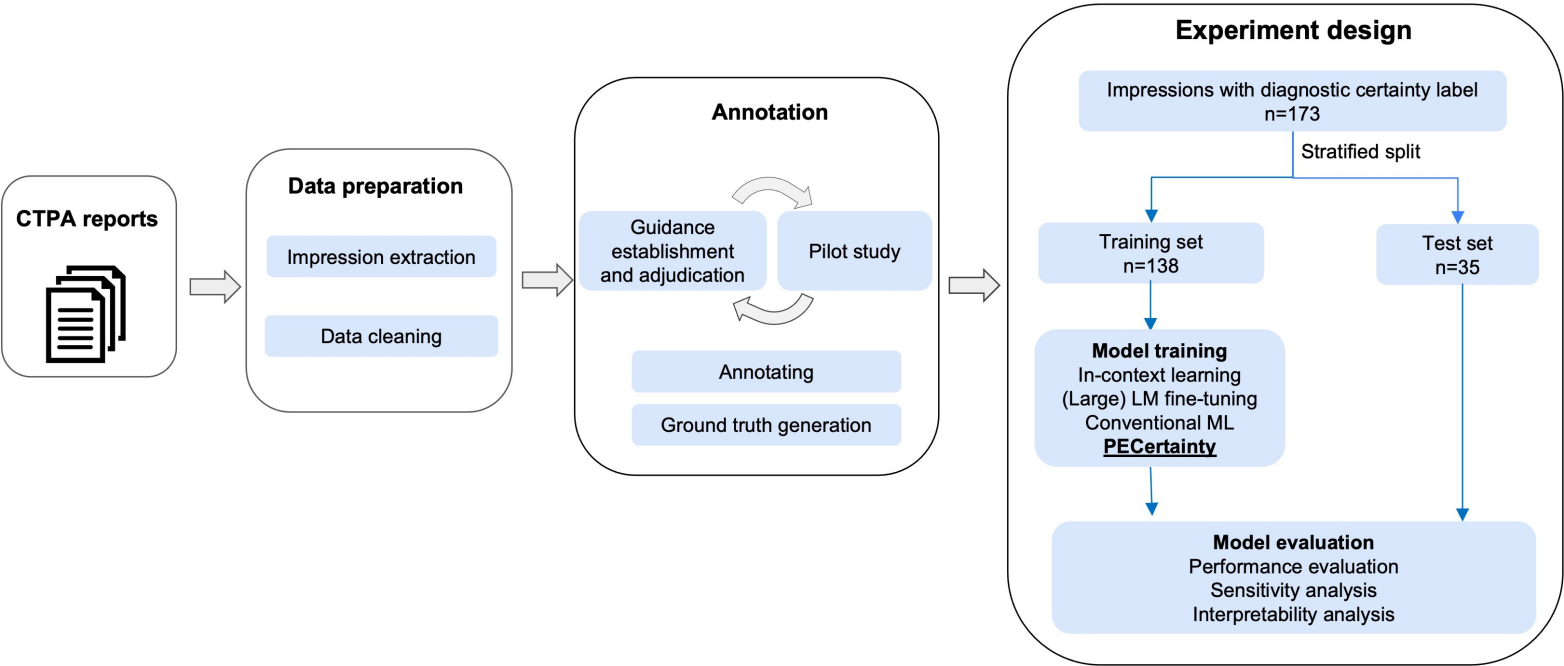
Overview of our study design. Data from October 1, 2017, to November 5, 2021, were retrieved, and a total of 205 reports were sampled and preprocessed for this study. This study includes data preparation, annotation, and experiment design. CTPA: Computed tomography pulmonary angiography; LM: language model; ML: machine learning.

Figure 1 provides an overview of our study design. The CTPA reports for model training were retrieved from the UMass Memorial Health (UMMH) system using the Nuance M-Power tool and PE-related terms from January 10, 2017, to May 11, 2021 (see Multimedia Appendix 1 for data retrieval details). A total of 205 reports were extracted and converted to plain text. Then, impression sections were extracted using regular expressions. A total of 3 board-certified radiologists independently classified each impression into 1 of 5 certainty categories (Table 1) based on consensus-based annotation guidelines (Multimedia Appendix 1). To ensure high annotation quality, we used an iterative process to refine guidelines and enhance interannotator agreement. Due to class imbalance, we merged the probable positive and probable negative categories into a single “probable” certainty category and excluded nondiagnostic examples. This adjustment resulted in 173 annotated notes for this study: definitive negative (n=106), probable (n=35), and definitive positive (n=32). We performed a stratified data split, using 80% (n=138) for training and 20% (n=35) for testing. Note that in our dataset, each report belongs to a unique patient, so report- and patient-level data splits are equivalent.

**Table 1.** Diagnostic certainty of diagnosis in the impression section of a radiology report—categories for annotation.

Certainty categories	Interpretation	Example of impression section (abridged)
Definitive positive	Describing discrete diagnostic positive findings with no hedging (>90% confidence) words.	Bilateral pulmonary embolism originating within the distal main pulmonary arteries and extending up to subsegmental levels…
Definitive negative	Describing discrete diagnostic findings with no hedging (>90% confidence) words.	No evidence of pulmonary embolism. Improving ground glass opacities, likely sequela of prior COVID pneumonia…
Probable positive	Containing contextual information indicating uncertainty with hedging or other differential diagnoses or mentioning suboptimal examination, although “PE”^a^ or “evidence of PE” may be mentioned.	There is a low-density filling defect in a segmental pulmonary artery branch, which may represent an embolus but could also be an artifact.
Probable negative	Containing contextual information indicating uncertainty with hedging or other differential diagnoses or mentioning involved suboptimal examination, although “no PE” or “no evidence of PE” may be mentioned.	Mildly limited study due to underopacification of the pulmonary arteries. Within this limitation, there is no evidence for pulmonary embolism up to the lobar branches…
Nondiagnostic	Containing an explicit description of being nondiagnostic due to technical and/or patient-related limitations.	The bolus of contrast is concentrated in the thoracic aorta and not in the pulmonary arteries. The examination is non-diagnostic for the evaluation of emboli of the pulmonary arteries. If there is a concern for pulmonary embolus, consider repeat…

a PE: pulmonary embolism.

We compared PECertainty against competitive baselines in terms of classification performance and conducted sensitivity analyses across varying training data sizes. To evaluate generalizability, we tested PECertainty on an external dataset of 420 reports from Baystate Medical Center, annotated using a different guideline: definitive (n=356; combining definitive positive and negative) and probable (n=64). Additionally, we performed interpretability analysis to assess the transparency and explainability of PECertainty. The demographic information of the datasets can be found in Multimedia Appendix 1.

### PECertainty

**Figure 2.**
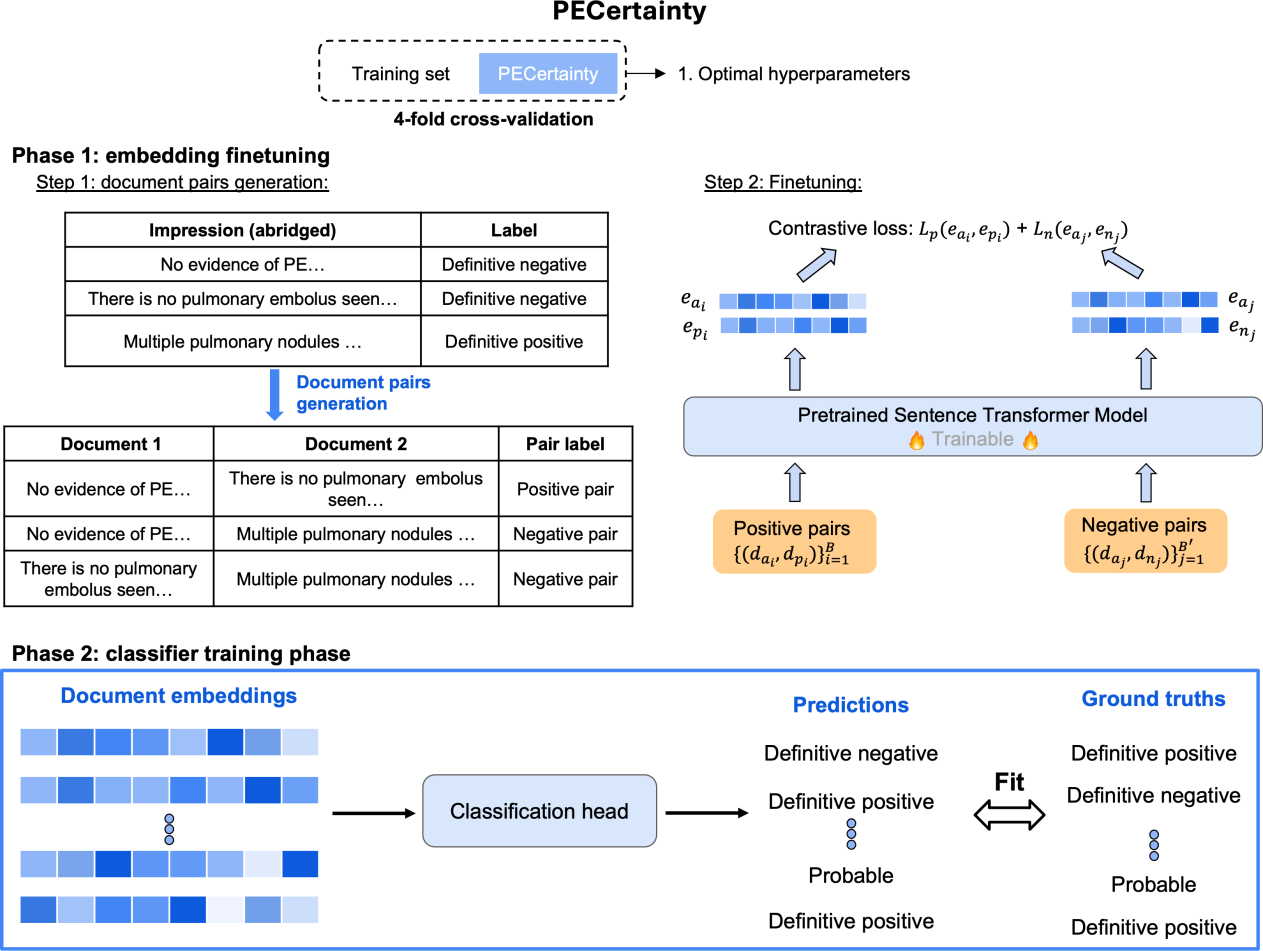
A total of 2-phase training framework of PECertainty. The framework consists of 2 phases. In phase 1, positive and negative document pairs are generated based on diagnostic certainty labels and used to fine-tune a pretrained sentence transformer using contrastive loss. In phase 2, sentence embeddings from the fine-tuned model are used to train a classification head for diagnostic certainty prediction. The hyperparameters are decided based on 4-fold cross-validation. PE: pulmonary embolism.

We developed PECertainty, a prompt-free data-efficient model for assessing diagnostic certainty of PE findings in radiology reports, leveraging the SetFit framework [19]. Figure 2 illustrates the PECertainty development pipeline, which consists of 2 key phases.

In phase 1, we generate sentence pairs for contrastive learning by sampling positive pairs from sentences belonging to the same diagnostic certainty class and negative pairs from sentences across different classes. These pairs are used to fine-tune the pretrained sentence transformer model “all-roberta-large-v1,” chosen for its strong empirical performance in sentence embedding tasks [21], using a contrastive loss based on cosine similarity. This process yields task-specific embeddings that encode subtle distinctions in diagnostic certainty classes.

In phase 2, we use the learned embeddings as input to a classification head, which is trained to predict 1 of the 3 certainty categories: definitive negative, probable, and definitive positive. This 2-phase training approach allows PECertainty to learn semantically meaningful and diagnostically aligned representations with minimal supervision.

### Baselines

We compare the performance of PECertainty against prompt-free and prompt-dependent methods. Prompt-free methods include conventional machine learning models, SVM [22], and random forest [23] integrated with the BioWordVec embedding model, as well as fine-tuned transformer-based models: RoBERTa-large, BioBERT (Bidirectional Encoder Representations From Transformers for Biomedical Text Mining), and ClinicalBERT (Bidirectional Encoder Representations From Transformers Pretrained on Clinical Text). For prompt-dependent methods, we evaluated state-of-the-art approaches, including ADAPET [24]. We also assessed LLM–based methods, including GPT-3.5 (“gpt-3.5-turbo-0125”) fine-tuning and in-context learning [18], as well as Gemma3-4B and Llama3.2-3B in-context learning. For in-context learning, demonstration examples were ranked based on their similarity to the query instance.

### Model Implementation

**Figure 3.**
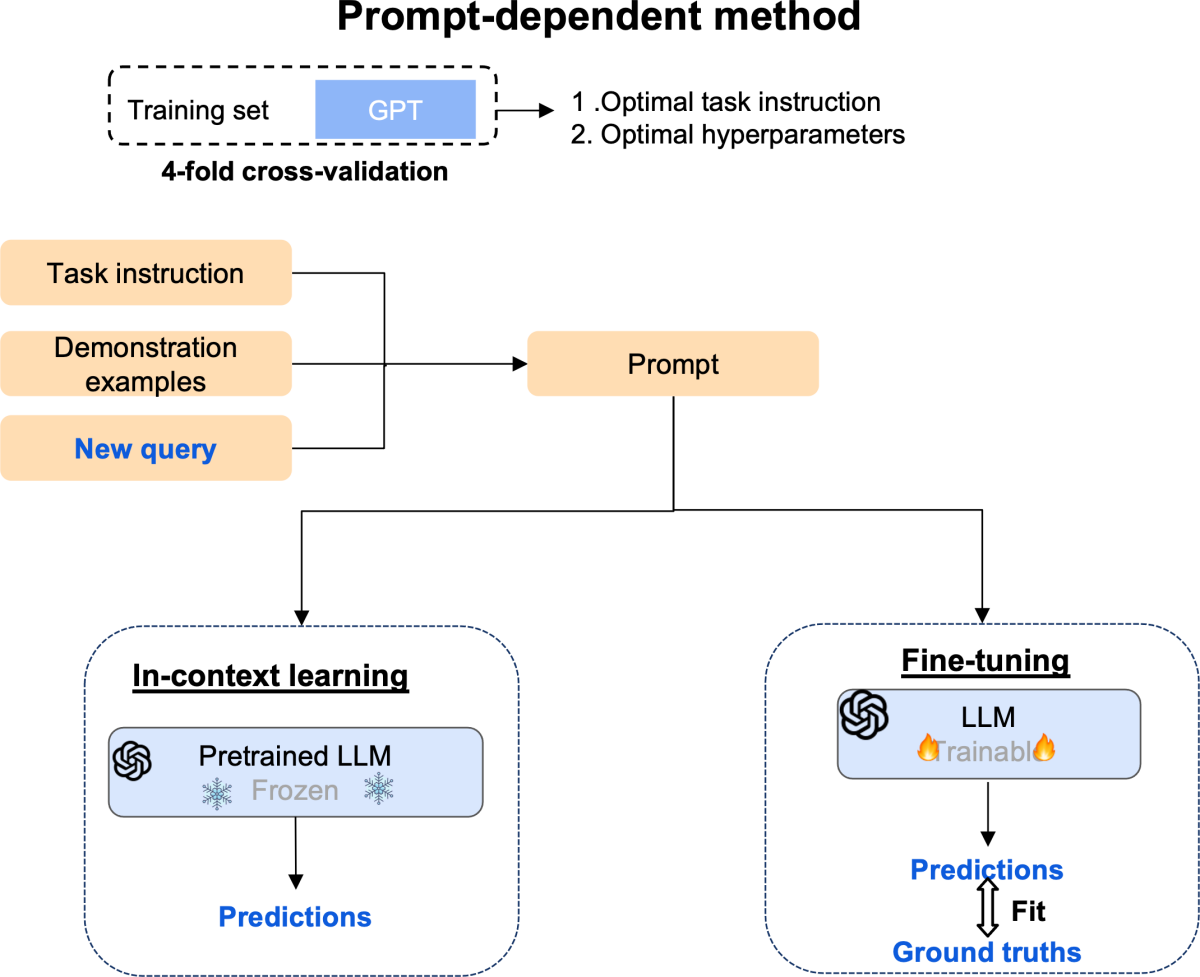
Pipeline of prompt-dependent method development. We select GPT-3.5 as the example to demonstrate the process. In in-context learning (left), a frozen pretrained LLM receives a prompt composed of task instructions, demonstration examples, and a new query and directly produces a prediction. In fine-tuning (right), an LLM is further trained on labeled data, optimizing model weights using a loss function between predictions and ground truth. LLM: large language model.

As shown in Figures 2 and 3, hyperparameter optimization was conducted with grid search and 4-fold cross-validation on the training data, which also applied to other methods (Multimedia Appendix 2). The prompt-free methods process the impression section, generate embeddings using various pretrained models, and feed these into a classifier for prediction. In contrast, the prompt-dependent model, such as GPT-3.5, combines task instructions, demonstration examples, and a query impression note to generate a PE diagnostic certainty label (Figure 3). Additional details are in Multimedia Appendix 2.

### Robustness Analysis

To assess the model’s robustness, we applied a k-shot learning setting, where *k* represents the number of labeled demonstration examples per class. We randomly sampled up to 10 examples per category from the training set and systematically varied *k* from 1 to 10. GPT-3.5 fine-tuning was restricted to 5 or more shots per category due to API requirements. Additionally, we evaluate the PECertainty trained on UMMH data on the external data from Baystate Medical Center to evaluate the generalization capability. As the Baystate annotation guideline differs from that of UMMH and defines only definitive and probable classes, the definitive positive and definitive negative predictions were combined into a single definitive category for external validation.

### Interpretability Analysis

**Figure 4.**
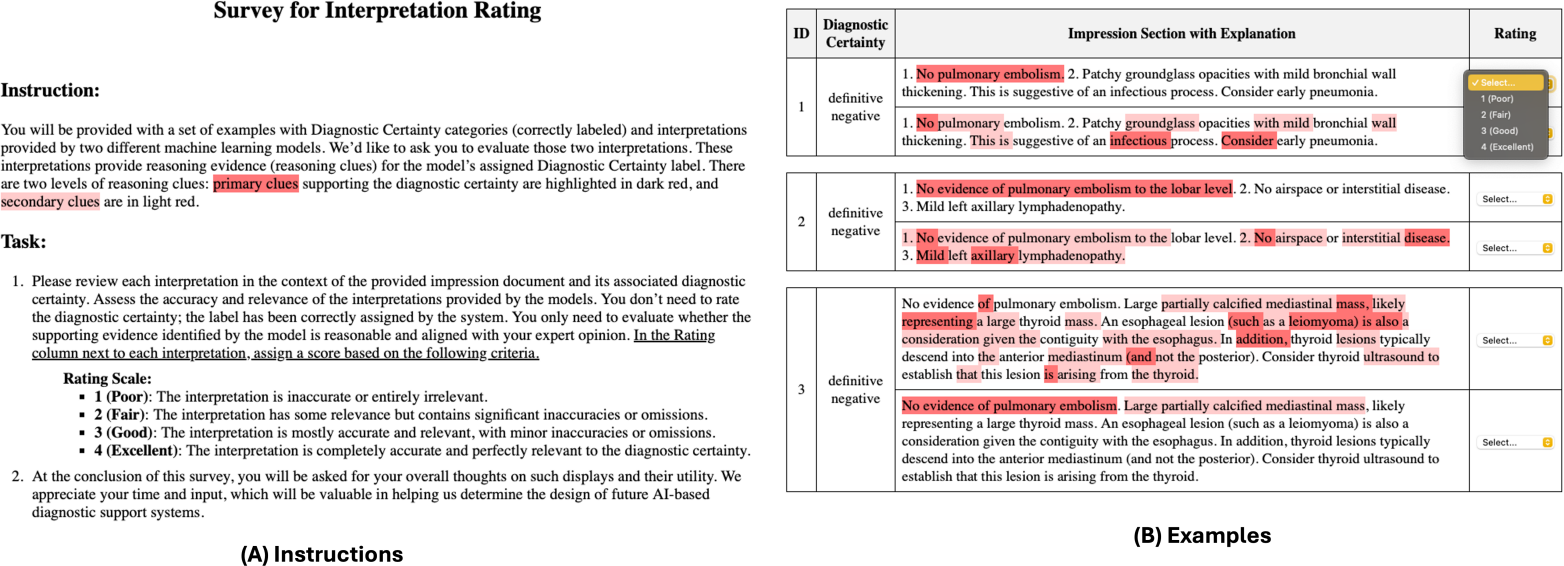
Screenshot of the interpretation rating survey. The primary clues supporting the diagnostic certainty are highlighted in dark red, and secondary clues are in light red. AI: artificial intelligence.

We also compared the interpretability of the 2 top-performing models, fine-tuned GPT-3.5 and PECertainty. Model interpretation aims to provide transparent reasoning for the model’s decision (ie, diagnostic certainty labels), which is important for clinical applications due to its high-stakes nature. For GPT-3.5, 0-shot prompting was used to prompt the model to identify primary and secondary clues based on the impression section and diagnostic certainty label (see Multimedia Appendix 3 for the prompt templates). For PECertainty, the integrated gradient method [21] was applied to calculate word importance scores. Words above the 75th percentile were considered primary clues, and those between the 50th and 75th percentiles were secondary clues. A total of 3 radiologists rated these clues on a 1‐4 scale (poor to excellent) using the criteria in Figure 4.

### Statistical Analysis

To evaluate radiologists’ agreement in categorizing impression notes, we used 3 statistics: Cohen [25], Fleiss [26], and Krippendorff [27]. We evaluated model performance using 5 standard metrics: accuracy, sensitivity, specificity, *F*_1_-score, and the area under the receiver operating characteristic curve, with 95% CIs calculated via 2000 times bootstrap resampling. Area under the curve (AUC) was not applicable for GPTs, as they provide token probabilities rather than probabilities for all categories. Overall performance was assessed using the macro-average, which averages metrics across categories.

For the interpretability analysis, we used the 2-tailed Wilcoxon signed-rank test [28] to compare the interpretability ratings of LLM and PECertainty from the same rater. This tests the null hypothesis of no significant difference between the ratings assigned to the 2 models by a given rater. Statistical analyses were conducted in Python (version 3.10; Python Software Foundation) using *scikit-learn***,** *krippendorff***,** *statsmodels***,** and *scipy* packages. To provide a more robust and statistically sound comparison, we employed a non-parametric bootstrap resampling procedure (B=1,000) to estimate confidence intervals for all evaluation metrics. In addition, we conducted paired bootstrap significance tests to assess performance differences between the baseline methods and PECertainty (B = 1,000). Statistical significance was determined using the bootstrap percentile method; results were considered statistically significant at the 0.05 level if the 95% confidence interval did not include zero.

## Results

### Human Annotator Agreement

Cohen for each pair of annotators is 0.81 (SD 0.038), 0.89 (SD 0.032), and 0.81 (SD 0.039). The mean of pair-wise Cohen is 0.84 (SD 0.030). The Fleiss score is 0.84 (SD 0.030), and the Krippendorff score is 0.85 (SD 0.030). These results collectively indicate a consistent agreement among the annotators with the guideline. We adjudicated all the annotation discrepancies based on the annotators’ consensus to serve as ground truth.

### Performance

The test set consisted of 35 reports, including 23 definitive negative, 6 probable, and 6 definitive positive. Table 2 summarizes the performance of all models on the withheld test set for PE certainty classification. Among the prompt-dependent methods, GPT-3.5 fine-tuning and in-context learning achieved *F*_1_-scores of 0.86 and 0.87, respectively, with no statistically significant difference between their performances (*t*paired bootstrap significance test, *P*>.05 for accuracy, recall, and specificity). Its 0-shot performance was lower (*F*_1_-score 0.57, 95% CI 0.40‐0.75). Overall, GPT-3.5 obtained better results than ADAPET (*F*_1_-score 0.42, 95% CI 0.32‐50). Gemma3-4B in-context learning (*F*_1_-score 0.63, 95% CI 0.56‐0.79) yields better performance than both its 0-shot setting (*F*_1_-score 0.45, 95% CI 0.29‐0.56) and the Llama3.2-3B under both in-context (*F*_1_-score 0.54, 95% CI 0.41‐0.71) and 0-shot (*F*_1_-score 0.43, 95% CI 0.28‐0.62) conditions.

**Table 2.** Model performance for certainty category prediction. “gpt-3.5-turbo-0125” in the table corresponds to the GPT-3.5 model in the main text. “Biow2v” denotes BioWordVec, which represents biomedical word embeddings generated using fastText. *P* values^a^ correspond to paired bootstrap significance test evaluating whether PECertainty’s performance differs significantly from that of each comparator model.

	Accuracy	Precision	Recall	*F*_1_-score	Specificity	AUC^b^
Prompt-free method						
SVM^c^ (Biow2v)	0.77^***a^ (0.63, 0.89)	0.69^***^ (0.50, 0.86)	0.68^***^ (0.5, 0.86)	0.67^***^ (0.49, 0.85)	0.86^***^ (0.77, 0.95)	0.81^***^ (0.67, 0.93)
RF^d^ (Biow2v)	0.71^***^ (0.57, 0.83)	0.68^***^ (0.39, 0.91)	0.57^***^ (0.40, 0.75)	0.59^***^ (0.39, 0.78)	0.77^***^ (0.68, 0.87)	0.79^***^ (0.64, 0.92)
Fine-tuning RoBERTa-large^e^	0.66^***^ (0.51, 0.8)	0.53^***^ (0.35, 0.72)	0.54^***^ (0.35, 0.73)	0.52^***^ (0.35, 0.71)	0.80^***^ (0.71, 0.89)	0.71^***^ (0.54, 0.85)
Fine-tuning BioBERT^f^	0.66^***^ (0.57, 0.70)	0.22^***^ (0.20, 0.25)	0.33^***^ (0.27, 0.40)	0.26^***^ (0.23, 0.30)	0.83^***^ (0.78, 0.90)	0.400^***^ (0.26, 0.54)
Fine-tuning ClinicalBERT^g^	0.92^*^ (0.83, 1.0)	0.91^***^ (0.83, 1.0)	0.88^***^ (0.75, 1.0)	0.87^***^ (0.71, 1.0)	0.95^***^ (0.90, 1.0)	0.99^***^ (0.97, 1.0)
PECertainty	0.94^h^ (0.86, 1.0)	0.97^h^ (0.94, 1.0)	0.89^h^ (0.72, 1.0)	0.92^h^ (0.79, 1.0)	0.98^h^ (0.95, 1.0)	0.99^h^ (0.97, 1.0)
Prompt-dependent method						
ADAPET^i^ (RoBERTa-large)	0.71^***^ (0.66, 0.77)	0.4^***^ (0.31, 0.47)	0.44^***^ (0.33, 0.56)	0.42^***^ (0.32, 0.5)	0.87^***^ (0.82, 0.91)	0.76^***^ (0.67, 0.84)
Gemma3-4B Zero-shot	0.71^***^ (0.66, 0.77)	0.57^***^ (0.26, 0.60)	0.44^***^ (0.33, 0.56)	0.45^***^ (0.29, 0.56)	0.90^***^ (0.87, 0.92)	N/A^j^
Gemma3-4B In-Context learning	0.68^***^ (0.54, 0.83)	0.68^***^ (0.52, 0.82)	0.42^***^ (0.57, 0.86)	0.63^***^ (0.56, 0.79)	0.84^***^ (0.79, 0.89)	N/A
Llama3.2-3B Zero-shot	0.69^***^ (0.60, 0.77)	0.53^***^ (0.26, 0.75)	0.43^***^ (0.30, 0.60)	0.43^***^ (0.28, 0.62)	0.85^***^ (0.78, 0.91)	N/A
Llama3.2-3B In-Context learning	0.68^***^ (0.57, 0.80)	0.61^***^ (0.43, 0.82)	0.63^***^ (0.54, 0.76)	0.54^***^ (0.41, 0.71)	0.84^***^ (0.79, 0.90)	N/A
gpt-3.5-turbo-0125 Zero-shot	0.77^***^ (0.66, 0.86)	0.67^***^ (0.37, 0.85)	0.6^*^ (0.43, 0.76)	0.57^***^ (0.4, 0.75)	0.9^***^ (0.82, 0.95)	N/A
gpt-3.5-turbo-0125 In-Context learning	0.91^*^ (0.83, 1.0)	0.90^*^ (0.83, 1.0)	0.87^*^ (0.73, 1.0)	0.87^*h^ (0.71, 1.0)	0.95^*^ (0.89, 1.0)	N/A
gpt-3.5-turbo-0125 Fine-tuning	0.92^*h^ (0.83, 1.0)	0.91^*h^ (0.83, 1.0)	0.88^*h^ (0.75, 1.0)	0.86^***^ (0.71, 1.0)	0.96^*h^ (0.90, 1.0)	N/A

a *P* value codes: * *P*≥.05; *** *P*<.05.

b AUC: area under the receiver operating characteristic curve.

c SVM: support vector machine.

d RF: random forest classifier

e RoBERTa-large: Robustly Optimized Bidirectional Encoder Representations from Transformers Pretraining Approach (Large Model).

f BioBERT: Bidirectional Encoder Representations From Transformers for Biomedical Text Mining.

g ClinicalBERT: Bidirectional Encoder Representations From Transformers for Clinical Text.

h PECertainty and GPT-3.5-turbo-0125 finetuning achieve similar performance and outperform other methods.

i ADAPET: A Densely-Supervised Approach to Pattern Exploiting Training.

j N/A: area under the receiver operating characteristic curve was not calculated for the large language model–based models because class labels were derived through prompt engineering, and token-level log-probabilities for predefined label strings were not extracted for area under the receiver operating characteristic curve calculation in this study.

PECertainty demonstrates numerically higher performance than all prompt-free methods, including fine-tuned ClinicalBERT (paired bootstrap significance test, *P*>.05 for accuracy and *P*<.05 across all other metrics), and exceeds the prompt-dependent ADAPET by a large margin. Furthermore, it is comparable to GPT-3.5 in both in-context learning and fine-tuning settings (paired bootstrap significance test, *P*<.05 for all metrics). It achieved the best *F*_1_-score of 0.92 (95% CI 0.79‐1) and the best AUC of 0.99 (95% CI 0.97‐1), competitively comparable with GPT3.5, yet it has a ≈500 times smaller (355M) parameter size than GPT3.5 (175B). Note that the significance values reported here could be sensitive to small changes in classification outcomes due to limited sample size, so the reported differences may not be definitive statistical evidence.

**Figure 5.**
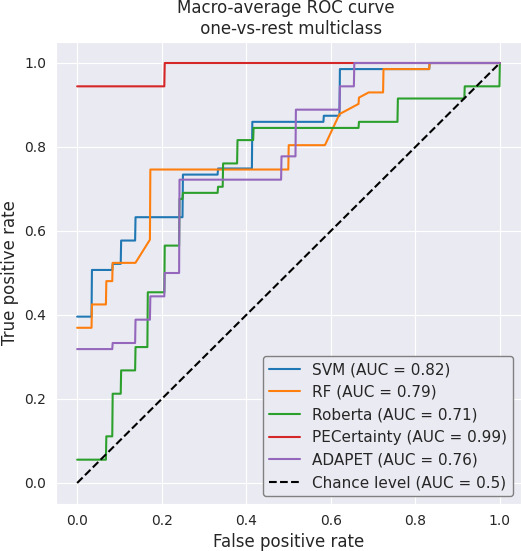
ROC curve of all methods (excluding LLMs). ADAPET: A Densely-Supervised Approach to Pattern Exploiting Training; AUC: area under the receiver operating characteristic curve; LLM: large language model; RF: random forest; ROC: receiver operating characteristic; SVM: support vector machine; RoBERTa: Robustly Optimized Bidirectional Encoder Representations from Transformers Pretraining Approach.

The misclassified examples and error analysis can be found in Multimedia Appendix 4. While all fine-tuned BERT variants benefit from pretraining, PECertainty consistently yields better performance than ClinicalBERT and BioBERT, suggesting that the additional embedding fine-tuning and task-specific alignment training objective of PECertainty contribute to improved performance. We also found that fine-tuning BioBERT and RoBERTa-large (Robustly Optimized Bidirectional Encoder Representations from Transformers Pretraining Approach [Large Model]) failed to outperform the conventional machine learning models of SVM and RF (*F*_1_-score of 0.26‐0.52 vs 0.59‐0.67 and AUC of 0.40‐0.71 vs 0.79‐0.81) when only limited training data is available. In contrast, ClinicalBERT fine-tuning achieved a substantially higher *F*_1_-score of 0.87, outperforming other pretrained models such as RoBERTa-large and BioBERT, thereby highlighting the importance of domain-specific data used during pretraining. RoBERTa-large is not pretrained with biomedical or clinical data. Both BioBERT and ClinicalBERT originate from the BERT architecture but were continually adapted on distinct domain corpora: BioBERT on biomedical literature from PubMed and PubMed Central (PMC), and ClinicalBERT on clinical narratives from the MIMIC-III (Medical Information Mart for Intensive Care III) database, including radiology and discharge notes. As our dataset exhibits linguistic patterns and contextual cues similar to clinical documentation, ClinicalBERT is better aligned with the task, which likely accounts for its superior performance relative to BioBERT. These performance differences can therefore be attributed primarily to variations in the domain-specific data used during pretraining. Figure 5 displays the receiver operating characteristic curves for all methods.

### Robustness

For the k-shot setting, Table 3 summarizes the performance of the best 3 methods with k examples per category, with k varying from 10 to 1. As the test set is class-imbalanced, the *F*_1_-score is a more informative metric for interpreting the results. For in-context learning, the *F*_1_-score is 0.57 in the 0-shot setting and peaks at 5 shots per category with an *F*_1_-score of 0.85. It then decreases as more shots per category are added, ending at 0.65 for 10 shots per category. The fine-tuned gpt-3.5-turbo-0125 outperforms its in-context learning counterpart with 10 and 7 shots per category, which suggests that while in-context learning can yield rapid gains in performance, fine-tuning can lead to better overall results when there are more examples available. The performance of PECertainty and GPT-3.5 fine-tuning and in-context learning declined in the 10-shot setting, likely reflecting the diminishing utility of additional examples once the most informative demonstrations had been included. As more examples were added, the additional cases were less semantically similar and introduced variability or noise that may have distracted the model from the primary task. Future work should focus on strategies for identifying and selecting high-value demonstrations so that additional examples enhance, rather than degrade, model performance.

**Table 3.** Model performance of PE^a^ diagnostic certainty classification on the test set under the k-shot setting. Unless otherwise noted, data are mean values, with 95% CIs in parentheses.

k	Accuracy	Precision	Recall	*F*_1_-score	Specificity	AUC^b^
gpt-3.5-turbo-0125 In-context learning						
10	0.77 (0.63, 0.89)	0.72 (0.46, 0.87)	0.72 (0.59, 0.86)	0.65 (0.47, 0.83)	0.88 (0.82, 0.94)	N/A^c^
7	0.8 (0.66, 0.91)	0.82 (0.78, 0.89)	0.81 (0.67, 0.94)	0.76 (0.6, 0.89)	0.88 (0.83, 0.94)	N/A
5	0.91^d^ (0.83, 1.0)	0.89 (0.83, 1.0)	0.87^d^ (0.73, 1.0)	0.85^d^ (0.69, 1.0)	0.95 (0.9, 1.0)	N/A
3	0.89 (0.8, 0.97)	0.86^d^ (0.75, 0.95)	0.78 (0.61, 0.94)	0.78 (0.58, 0.94)	0.96^d^ (0.92, 0.99)	N/A
1	0.8 (0.71, 0.89)	0.73 (0.45, 0.94)	0.7 (0.61, 0.82)	0.65 (0.5, 0.84)	0.89 (0.81, 0.96)	N/A
0	0.77 (0.66, 0.86)	0.67 (0.37, 0.85)	0.6 (0.43, 0.76)	0.57 (0.4, 0.75)	0.9 (0.82, 0.95)	N/A
gpt-3.5-turbo-0125 Fine-tuning						
10	0.77 (0.63, 0.89)	0.77 (0.63, 0.87)	0.8 (0.66, 0.93)	0.74 (0.58, 0.88)	0.87 (0.82, 0.93)	N/A
7	0.83^d^ (0.69, 0.94)	0.84^d^ (0.78, 0.92)	0.83^d^ (0.69, 0.96)	0.79^d^ (0.63, 0.91)	0.9^d^ (0.84, 0.96)	N/A
5	0.71 (0.57, 0.86)	0.79 (0.76, 0.85)	0.77 (0.63, 0.9)	0.71 (0.56, 0.84)	0.85 (0.8, 0.9)	N/A
PECertainty						
10	0.89 (0.8, 0.97)	0.87 (0.75, 0.97)	0.78 (0.61, 0.94)	0.80 (0.59, 0.94)	0.92 (0.85, 0.99)	0.91 (0.82, 0.99)
7	0.89 (0.8, 0.97)	0.9 (0.77, 0.99)	0.78 (0.61, 0.94)	0.81 (0.6, 0.96)	0.91 (0.82, 0.98)	0.95^d^ (0.88^d^, 0.99^d^)
5	0.91^d^ (0.83, 1.0)	0.96^d^ (0.93, 1.0)	0.83 (0.67, 1.0)	0.87^d^ (0.73^d^, 1.0^d^)	0.92 (0.83, 1.0)	0.88 (0.74, 1.0)
3	0.51 (0.37, 0.66)	0.36 (0.27, 0.44)	0.38 (0.23, 0.54)	0.34 (0.24, 0.44)	0.77 (0.69, 0.85)	0.66 (0.51, 0.8)
1	0.52 (0.4, 0.63)	0.23 (0.2, 0.25)	0.26 (0.2, 0.32)	0.24 (0.21, 0.28)	0.68 (0.61, 0.73)	0.59 (0.46, 0.71)

a PE: pulmonary embolism.

b AUC: area under the receiver operating characteristic curve.

c N/A: area under the receiver operating characteristic curve was not calculated for the large language model–based models because class labels were derived through prompt engineering, and token-level log-probabilities for predefined label strings were not extracted for area under the receiver operating characteristic curve calculation in this study.

d Best performance for each metric.

On the Baystate Medical Center dataset, PECertainty achieved an accuracy of 0.90 (95% CI 0.87‐0.92), precision of 0.82 (95% CI 0.76‐0.89), recall of 0.73 (95% CI 0.67‐0.79), and an *F*_1_-score of 0.77 (95% CI 0.70‐0.83). The model also demonstrated strong specificity (0.82, 95% CI 0.76‐0.89) and AUC of 0.84 (95% CI 0.77‐0.90). Notably, because the external Baystate dataset merges positive and negative findings into a single definitive category, the reported metrics evaluate the model’s ability to distinguish certainty from uncertainty rather than its capacity for diagnostic polarity classification. As the external dataset is highly imbalanced with substantially more definitive cases than probable cases, we also reported class-specific performance to characterize the model generalization. For the minority “probable” class (n=64), PECertainty achieved a precision of 0.73 (95% CI 0.61‐0.85), recall of 0.50 (95% CI 0.38‐0.63), and *F*_1_-score of 0.60 (95% CI 0.48‐0.80). These results demonstrate that the model identifies a meaningful proportion of uncertain cases and performs substantially better than a naive classifier that predicts only the majority definitive class. For the definitive class (n=356), PECertainty achieved a precision of 0.92 (95% CI 0.90‐0.94), recall of 0.97 (95% CI 0.95‐0.98), and *F*_1_-score of 0.94 (95% CI 0.93‐0.95). While performance naturally varies across classes due to sample size differences, these results indicate that the model generalizes well to the external dataset. Specifically, it maintains strong discriminative ability in distinguishing certain from uncertain cases, even in the presence of class imbalance.

### Interpretability

The distributions of the ratings from 3 raters are shown in Figure S1 in Multimedia Appendix 3. The ratings for each impression section are shown in Figure S2 in Multimedia Appendix 3. We examined the differences in ratings provided by the 3 raters, using the average rating value as the overall rating for the interpretations generated by the LLM and the integrated gradient of PECertainty. As shown in Table 4, the Wilcoxon signed-rank test indicates that each of the 3 raters assigned significantly different ratings to LLM and PECertainty. The interpretations generated by LLMs are preferred. Some examples of the interpretations and their corresponding average ratings are shown in Table S4 in Multimedia Appendix 3.

**Table 4.** Results of the Wilcoxon signed-rank test. Wilcoxon signed-rank test statistic (W), and a smaller W indicates a larger difference between the 2 samples, suggesting that there is a significant difference between the paired observations.

	Wilcoxon signed-rank test statistic	*P* value
Rater 1	12.50	7.00 × 10^-4^
Rater 2	3.50	5.17 × 10^-5^
Rater 3	9.00	5.59 × 10^-5^

## Discussion

### Principal Findings

Our study demonstrates that the open-source, prompt-free PECertainty model achieves strong performance compared to both prompt-free and prompt-dependent baselines in assessing PE diagnostic certainty. With approximately 500 times fewer parameters, it yields comparable performance to the closed-source LLM GPT-3.5 when limited annotated data is available. Notably, GPT-3.5 in-context learning performs similarly to fine-tuning while being training-free and more efficient. Our experiments show that PECertainty outperforms ClinicalBERT fine-tuning, but it is noted that the performance comparison between PECertainty (SetFit+ RoBERTa) and the ClinicalBERT baseline may be confounded by differences in training strategy, as ClinicalBERT was not fine-tuned using the SetFit framework. In the k-shot setting, PECertainty benefits from a few examples per category but shows limited improvement with larger datasets. It obtained better performance than GPT-3.5 (gpt-3.5-turbo-0125) in both fine-tuning and in-context learning with more than 5 shots per category, highlighting its strong few-shot learning capability. External validation further supports the model’s generalizability for certainty classification, although this evaluation does not assess diagnostic polarity because definitively positive and definitively negative reports are aggregated in the external dataset.

For interpretability analysis, the explanations generated by the LLM were preferred over those produced by PECertainty (*t* test, P<.05). This finding highlights that the quality of model interpretability directly influences the usability of AI tools in clinical practice. Although PECertainty achieves strong classification performance and provides a faithful, model-grounded explanation through integrated gradients, its continuous attribution maps may be less intuitive for radiologists to interpret during routine workflow. While integrating LLM-generated explanations of the PECertainty predictions could improve clinician trust based on our findings, this approach would require deploying both PECertainty and a large LLM simultaneously, thereby increasing computational cost and reducing inference efficiency. Until interpretability is further improved, PECertainty is best deployed as a backend triage and worklist prioritization tool, rather than as a direct clinician-facing decision-support system. In this role, the model can assist by flagging high-risk cases for expedited review while minimizing the risk of clinician confusion or overreliance on the model outputs.

Despite overall strong performance, PECertainty revealed rare but clinically significant failure modes (shown in Multimedia Appendix 4), for example, polarity inversion error by misclassifying a clearly positive statement as “definitive negative” due to contextual ambiguity. This represents a catastrophic negation error with potentially serious patient safety implications for high-risk conditions such as PE, as it could lead to inappropriate clinical reassurance if no safeguards were added. It highlights limitations of end-to-end language models in reliably handling heterogeneous contexts. Therefore, the system should not be used as an autonomous diagnostic tool as the sole basis for ruling out disease. Different mitigation strategies can be explored for future safe deployment. First, hybrid strategies can be implemented to combine data-driven learning with rule-based guardrails, which can identify typical linguistic patterns to help reduce polarity inversion errors. Second, confidence-based thresholds can be used to automatically route ambiguous cases for mandatory radiologist review. Third, a targeted data augmentation in training can be applied to improve the model’s sensitivity to this specific type of error. We emphasize that any clinical deployment of this system would require robust human oversight, with radiologists retaining full responsibility for diagnostic decisions. This human-in-the-loop paradigm helps mitigate the impact of catastrophic errors while preserving the efficiency benefits of automated assistance.

### Comparison to Prior Work

The advent of picture archiving and communication system [29] has reduced direct communication between radiologists and referring physicians, making clear diagnostic certainty in reports essential. However, inconsistent interpretation of hedging phrases often leads to ambiguity, delaying treatments or causing unnecessary interventions. Standardized lexicon in hedging [6] fails to capture contextual certainty semantics. Addressing these challenges calls for a standardized, context-aware uncertainty measurement. Yet existing rule-based approaches [17] rely on labor-intensive linguistic indicators, hindering scalability and omitting probabilistic assessments. This method also overlooks nuanced contextual interactions between disease status (eg, positive vs negative) and certainty. In contrast, machine learning techniques, particularly deep learning, can automate and enhance the quantification of diagnostic uncertainty. While traditional models (eg, SVMs and random forests) use word embeddings (eg, BioWordVec), they lack deeper contextual understanding. Fine-tuning PLMs has become a state-of-the-art practice for capturing context, but it usually requires substantial labeled data. When labeled data is scarce, few-shot learning frameworks built on PLMs offer a compelling alternative. The PECertainty was adapted from the SetFit approach and demonstrated its effectiveness and improved data efficiency in assessing diagnostic uncertainty of CTPA reports.

The PECertainty model offers 2 primary advantages. First, unlike prompt-dependent few-shot models, it eliminates the need for manually crafted verbalizers or prompts. Models such as ADAPET require cloze-style phrases and label-word mappings, while GPT-based methods rely on task descriptions, introducing variability from manual engineering. Instead, PECertainty generates rich embeddings from a small set of labeled examples. Second, PECertainty does not require large-scale pretrained models to achieve high performance. While LLMs such as GPT contain billions of parameters, PECertainty leverages smaller models, making training and inference significantly faster. However, interpretability analysis showed that GPT-3.5’s explanations were rated higher than PECertainty’s gradient-based interpretations.

PECertainty offers a scalable and efficient approach to standardizing diagnostic certainty in radiology reports. By providing referring physicians with clear, structured certainty labels (ie, definitive positive, definitive negative, and probable), the model can reduce ambiguity and support more consistent clinical decision-making. This standardization may help address known issues such as judgment variability among radiologists [30] and improve communication between radiologists and treating physicians. Additionally, once PECertainty is further improved on its interpretability, it could be integrated into clinical workflows as a decision-support tool within picture archiving and communication systems or structured reporting platforms. In this setting, the model could automatically analyze draft or finalized CTPA reports in real time to flag low-certainty impressions for review, promote clarification before report finalization, and alert clinicians to cases that warrant additional follow-up. Such integration could reduce unnecessary hedging, streamline communication, and help avoid treatment delays. In turn, this may help avoid delays in treatment or unnecessary anticoagulation. Future studies can build on this work to evaluate the association between report uncertainty and various clinical outcomes [31] as well as examine variation in report quality by health organizations, radiologists, and patient subgroups.

### Limitations

There are limitations to this study. First, although there are a lot of pretrained models in the RoBERTa and BERT families, we did not exhaustively compare all variants. We chose RoBERTa-large because it achieved excellent performance in many NLP benchmarking tasks [32] and has been further pretrained as a sentence transformer, making it more comparable to the model architecture used in PECertainty. In contrast, not all RoBERTa variants have publicly available pretrained sentence transformer versions suitable for direct comparison. BioBERT and ClinicalBERT were included for their domain-specific training on biomedical and clinical texts, respectively. Second**,** the dataset used in this study was modest in size (173 CTPA reports), which may influence model stability and generalizability. Nevertheless, PECertainty was specifically designed to operate under low-resource conditions, and its performance was further evaluated on an external Baystate Medical Center dataset to provide preliminary evidence of generalizability beyond the development cohort. Although the results are encouraging, they are interpreted as preliminary and warrant validation in larger, multi-institutional studies. Third, due to the scarcity of data belonging to the probable negative and probable positive classes, we merged the data from these 2 classes into 1 class labeled as “probable.” This consolidation sacrifices the modeling granularity with reduced clinical utility. In other words, the model cannot distinguish cases that convey high diagnostic concern (probable positive) from those reflecting lower suspicion (probable negative), which might lead to ambiguity in downstream decision-making. We plan to explore self-learning and active learning strategies to enrich the dataset with more “probable” examples, thereby enhancing the model’s ability to capture subtle clinical nuances in PE diagnostic communication and ultimately advance PE care management.

Fourth**,** the external validation dataset from Baystate Medical Center was annotated using a slightly different guideline, defining only definitive and probable categories, whereas our primary model was trained on 3 classes (definitive positive, definitive negative, and probable). The annotation bias and inconsistencies would be minimal because both teams applied conceptually aligned definitions and guidelines. However, to align these schemes for external validation, we had to combine our 2 “definitive” predictions into 1 category. This limited the scope of our external validation, that is, only validating the model’s ability to distinguish “certain” from “uncertain” reports on external data. More validation is warranted when external data with more refined annotations is available to test the model’s capacity in separating “definitive positive” reports from “definitive negative” ones. Fifth, the catastrophic negation errors (Multimedia Appendix 4) noted in the previous discussion indicate potential safety concerns. Therefore, the current iteration of PECertainty must be patched with optimal mitigation strategies before clinical deployment (eg, rule-based guardrails or low-confidence triggered mandatory expert review needs to be implemented and extensively evaluated). Sixth, research findings are time-limited due to the rapid evolution of LLMs and the continuous release of newer models, although newer models do not always mean improvement for a certain task [33]. Future investigators should replicate and extend our study using newer versions of LLMs. To address this limitation, future studies should replicate and extend our findings using newer versions of the LLMs evaluated here. Moreover, PECertainty is designed to be adaptable and modular, allowing integration of newer base models as they become available.

### Future Direction

For future work, we would focus on collecting additional cases in the probable category through active learning and developing models that can distinguish between the finer-grained classes (eg, probable negative vs probable positive). Moreover, expanding the evaluation to include a broader range of LLMs and exploring multitask learning (with additional subtasks such as PE acuity and location) may further enhance the model’s overall performance. Finally, we will explore effective and innovative ways to improve PECertainty’s interpretability that include (1) developing extractive rationalization mechanisms that identify and surface the specific textual evidence spans supporting each certainty classification; (2) exploring hybrid explainer approaches, such as teacher-student frameworks in which a constrained language model generates structured natural-language justifications aligned with PECertainty’s outputs; and (3) adopting a human-centered co-design strategy as a core component of future development. We plan to work closely with pulmonary doctors in the ED setting and CTPA radiologists to iteratively evaluate which forms of model evidence are most interpretable and actionable in high-pressure clinical environments.

### Conclusion

We developed PECertainty and evaluated baseline methods for assessing diagnostic certainty in CTPA reports under limited annotated data conditions. Our results demonstrate that PECertainty effectively captures the contextual semantics of free-text reporting to assess diagnostic certainty, even with scarce data. This holds the potential to facilitate precise communication of imaging findings between radiologists and referring physicians.

## Supplementary material

10.2196/79972 Multimedia Appendix 1 Data and annotating.

10.2196/79972 Multimedia Appendix 2 Implementation details.

10.2196/79972 Multimedia Appendix 3 Interpretability analysis.

10.2196/79972 Multimedia Appendix 4 Error analysis.

## Abbreviations

ADAPET: A Densely-Supervised Approach to Pattern Exploiting Training
AUC: area under the curve
BioBERT: Bidirectional Encoder Representations From Transformers for Biomedical Text Mining
ClinicalBERT: Bidirectional Encoder Representations From Transformers Pretrained on Clinical Text
CTPA: computed tomography pulmonary angiography
ED: emergency department
LLM: large language model
MIMIC-III: Medical Information Mart for Intensive Care III
NLP: natural language processing
PE: pulmonary embolism
PLM: pretrained language model
RoBERTa: Robustly Optimized Bidirectional Encoder Representations From Transformers Pretraining Approach
RoBERTa-large: Robustly Optimized Bidirectional Encoder Representations From Transformers Pretraining Approach (Large Model)
SVM: support vector machine
UMMH: UMass Memorial Health

## References

[1] Gunn AJ, Tuttle MC, Flores EJ (2016). Differing interpretations of report terminology between primary care physicians and radiologists. J Am Coll Radiol.

[2] Rosenkrantz AB (2017). Differences in perceptions among radiologists, referring physicians, and patients regarding language for incidental findings reporting. AJR Am J Roentgenol.

[3] Gunn AJ, Sahani DV, Bennett SE, Choy G (2013). Recent measures to improve radiology reporting: perspectives from primary care physicians. J Am Coll Radiol.

[4] Goldberg-Stein S, Chernyak V (2019). Adding value in radiology reporting. J Am Coll Radiol.

[5] Lafortune M, Breton G, Baudouin JL (1988). The radiological report: what is useful for the referring physician?. Can Assoc Radiol J.

[6] Panicek DM, Hricak H (2016). How sure are you, doctor? A standardized lexicon to describe the radiologist’s level of certainty. AJR Am J Roentgenol.

[7] Weiss DL, Langlotz CP (2008). Structured reporting: patient care enhancement or productivity nightmare?. Radiology.

[8] Shea LAG, Towbin AJ (2019). The state of structured reporting: the nuance of standardized language. Pediatr Radiol.

[9] Plumb AAO, Grieve FM, Khan SH (2009). Survey of hospital clinicians’ preferences regarding the format of radiology reports. Clin Radiol.

[10] Bosmans JML, Weyler JJ, De Schepper AM, Parizel PM (2011). The radiology report as seen by radiologists and referring clinicians: results of the COVER and ROVER surveys. Radiology.

[11] Danton GH (2010). Radiology reporting: changes worth making are never easy. Appl Radiol.

[12] Pons E, Braun LMM, Hunink MGM, Kors JA (2016). Natural language processing in radiology: a systematic review. Radiology.

[13] Fink MA, Kades K, Bischoff A (2022). Deep learning-based assessment of oncologic outcomes from natural language processing of structured radiology reports. Radiol Artif Intell.

[14] Bhayana R (2024). Chatbots and large language models in radiology: a practical primer for clinical and research applications. Radiology.

[15] Yan A, McAuley J, Lu X (2022). RadBERT: adapting transformer-based language models to radiology. Radiol Artif Intell.

[16] Liu F, Zhou P, Baccei SJ (2021). Qualifying certainty in radiology reports through deep learning–based natural language processing. AJNR Am J Neuroradiol.

[17] Chapman BE, Lee S, Kang HP, Chapman WW (2011). Document-level classification of CT pulmonary angiography reports based on an extension of the ConText algorithm. J Biomed Inform.

[18] Brown TB, Mann B, Ryder N (2020). Language models are few-shot learners. arXiv.

[19] Tunstall L, Reimers N, Jo UES (2022). Efficient few-shot learning without prompts. https://neurips2022-enlsp.github.io/papers/paper_17.pdf.

[20] 45 CFR part 46. Code of Federal Regulations.

[21] Reimers N, Gurevych I (2019). Sentence-BERT: sentence embeddings using siamese BERT-networks. https://www.aclweb.org/anthology/D19-1.

[22] Schölkopf B (1998). SVMs - a practical consequence of learning theory. IEEE Intell Syst.

[23] Breiman L (2001). Random forests. Mach Learn.

[24] Tam D, Menon RR, Bansal M, Srivastava S, Raffel C (2021). Improving and simplifying pattern exploiting training. Assoc Comput Linguist.

[25] McHugh ML (2012). Interrater reliability: the kappa statistic. Biochem Med (Zagreb).

[26] Fleiss JL (1971). Measuring nominal scale agreement among many raters. Psychol Bull.

[27] Hayes AF, Krippendorff K (2007). Answering the call for a standard reliability measure for coding data. Commun Methods Meas.

[28] Wilcoxon F (1992). Breakthroughs in Statistics.

[29] Reiner B, Siegel E, Protopapas Z, Hooper F, Ghebrekidan H, Scanlon M (1999). Impact of filmless radiology on frequency of clinician consultations with radiologists. Am J Roentgenol.

[30] Jaramillo D (2022). Radiologists and their noise: variability in human judgment, fallibility, and strategies to improve accuracy. Radiology.

[31] Bedayat A, Sewatkar R, Cai T (2015). Association between confidence level of acute pulmonary embolism diagnosis on CTPA images and clinical outcomes. Acad Radiol.

[32] Lan Z, Chen M, Goodman S (2020). ALBERT: a LITE BERT for self-supervised learning of language representations. arXiv.

[33] Chen L, Zaharia M, Zou J (2024). How Is ChatGPT’s behavior changing over time?. Harvard Data Sci Rev.

[34] PECertainty. GitHub.

