# Supplementary material for: Data-Efficient Language Model for Assessing Pulmonary Embolism Diagnostic Certainty From Radiology Reports: Model Development and Validation Study
Source: JMIR Med Inform. 2026 Apr 28;14:e79972. doi: 10.2196/79972 (PMC13123884; doi:10.2196/79972)
Supplement: Multimedia Appendix 1 — Data and annotating. [file medinform-v14-e79972-s001.docx]

### **Data Retrieval**

The PE-related terms ‘emboli | embolism | PE | embolus | "pulmonary arterial" | "pulmonary artery" | "pulmonary arteries"’ were searched in the impression section, and the retrieved results were filtered using the exam code “IMG1266: CT CHEST PULMONARY EMBOLISM W CONTRAST” in the emergency department (ED) setting.

### **Guidelines**

**Certainty PE Annotation Schema and Guidelines**

1. **Task**: Annotate the PE diagnostic certainty category conveyed in CTPA reports (impression section) to develop a reference standard for training/testing an automatic certainty assessment system for PE diagnostic communication (ED setting).
2. **Annotation Schema (either acute or chronic PE will be considered POS)**
   1. Definitive PE positive
   2. Probable PE positive
   3. Definitive PE negative
   4. Probable PE negative
   5. Indeterminate
   6. Non-diagnostic
3. **Definitions**
   1. Definitive: very certain, usually discreet diagnostic finding with no hedging (>90% confidence).
   2. Probable Positive/Negative: Contain contextual information indicating uncertain with hedging or other differential diagnosis or mentioning involved suboptimal examination, although “No PE” or “No evidence of PE” may be mentioned.
      1. Example hedging expressions
         - suggestive of, probably, concerning for, concerned with, most likely, possibly, could be, appear, seems, possibility of, unlikely, likely, often, low chance, no obvious, can, could, may, might, doubtful, uncertain, presumably, grossly, felt to represent, question mark(?), question, essentially, no significant, unclear, nonspecific, compatible with, unremarkable, coincide with, rarely, almost certain
         - Removed from the list: no evidence of, consistent with
   3. Positive/Negative: baseline assumption (what the report aims to communicate)
   4. Indeterminate: only describing some findings, but the language doesn’t indicate that diagnostic interpretation pertains to PE
   5. Non-diagnostic: a term meaning non-diagnostic explicitly mentioned due to technical and/or patient-related issues
4. **Special cases**
   1. “No evidence of xx” and “No xx” will both be considered as “definitive negative” cases, if there is no other description falling under 4b (definitions above).
   2. “Can’t be excluded”, “can’t be completely excluded” (more on the negative baseline assumption)

### **Annotating Interface**


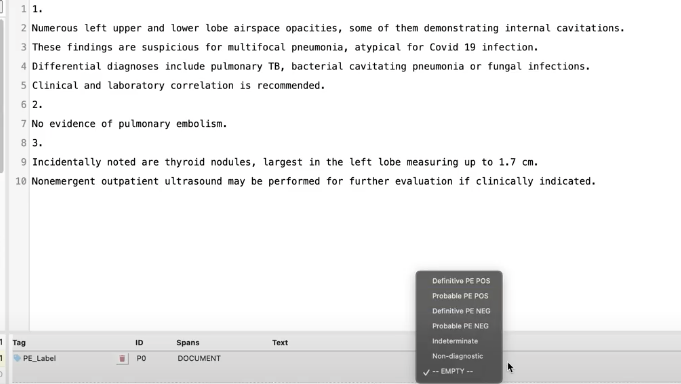


**Table S1.** UMMH data statistics.

|  |  | All | Definitive positive | Probable | Definitive negative |
| --- | --- | --- | --- | --- | --- |
| Sex | Male | 84 | 21 | 16 | 47 |
|  | Female | 89 | 11 | 19 | 59 |
| Age | <55 | 83 | 13 | 16 | 54 |
|  | 55-65 | 41 | 8 | 8 | 25 |
|  | >=65 | 49 | 11 | 11 | 27 |

**Table S2.** Baystate data statistics.

|  |  | All | Definitive | Probable |
| --- | --- | --- | --- | --- |
| Sex | Male | 182 | 147 | 35 |
|  | Female | 238 | 209 | 29 |
| Age | <55 | 90 | 76 | 14 |
|  | 55-65 | 80 | 69 | 11 |
|  | >=65 | 250 | 211 | 39 |
| Race | White | 334 | 286 | 48 |
|  | Not White | 86 | 70 | 16 |
