# Supplementary material for: Data-Efficient Language Model for Assessing Pulmonary Embolism Diagnostic Certainty From Radiology Reports: Model Development and Validation Study
Source: JMIR Med Inform. 2026 Apr 28;14:e79972. doi: 10.2196/79972 (PMC13123884; doi:10.2196/79972)
Supplement: Multimedia Appendix 2 — Implementation details. [file medinform-v14-e79972-s002.docx]

### **Prompt-dependent methods**

**ADAPET:** We fine-tuned the pre-trained RoBERTa-large model with learning rate =1e-5, weight_decay=1e-2, verbalizer = [‘negative’, ‘probable’, ’positive’]. The prompt is below:

'{text} Pulmonary embolism is [label]'.

**GPT-3.5, Gemma3-4B, Llama3.2-3B**:

We set the temperature to 0.1.

Prompt includes task instruction, demonstration examples, and query text:

*Task Instruction:* You are a radiologist. Please give a diagnostic certainty of pulmonary embolism based on the provided impressions section of the radiology report. Your job is to classify the provided document into one of the following labels: ['definitive negative', 'probable', 'definitive positive']. You will return the answer with just one element: 'the correct label'.

*Demonstration example: {text_1_} {label_1_};{text_2_} {label_2_};….{text_n_} {label_n_}*

*Query text: {text}*

### **Prompt-free methods**

**SVM:** The regularization parameter C=1, kernel type= ‘rbf’, gamma=0.1

RandomForest: criterion=’entropy’, max_depth =8, max_features=’sqrt’, min_sample_split=5, n_estimators=100

**RoBERTa fine-tuning:** epochs=6, learning rate=5e-06, batch_size=8

**PECertainty:** The hyperparameters used in phase 1 are as follows: learning rate = 2e-5, batch size = 16, number of iterations = 70. The hyperparameters used in phase 2 are as follows: learning rate = 2e-5, batch size = 16, epochs = 2.
