# Supplementary material for: Data-Efficient Language Model for Assessing Pulmonary Embolism Diagnostic Certainty From Radiology Reports: Model Development and Validation Study
Source: JMIR Med Inform. 2026 Apr 28;14:e79972. doi: 10.2196/79972 (PMC13123884; doi:10.2196/79972)
Supplement: Multimedia Appendix 3 — Interpretability analysis. [file medinform-v14-e79972-s003.docx]

The analysis is based on the GPT-3.5 model fine-tuned on all training data. We set the temperature to 0.1.

Prompt:

As a radiologist, you gave a diagnostic certainty of pulmonary embolism as one of the following labels: [definitive positive, definitive negative, probable] for the provided impression section. Firstly, identify the primary CLUES (limit to 10 words) from the document that support the diagnostic certainty of pulmonary embolism by surrounding the primary CLUES with '^'. Secondly, identify the secondary CLUES (limit to 10 words) from the document that support the diagnostic certainty of pulmonary embolism by surrounding the secondary CLUES with '@'.

Document:{impression section}, Label:{prediction}

Clues:

**Table S1**

**Table S1.** Examples of impression section with interpretations provided by different automated methods and the corresponding average ratings from three radiologists.

| **ID** | **Label** | **Method** | **Impression Section with Explanation** | **Average Rating** |
| --- | --- | --- | --- | --- |
| 1 | Definitive  Negative | LLM | 1. No evidence of pulmonary embolism. 2. No pneumonia. 3. Scattered left lung pulmonary nodules largest 6 mm, likely of doubtful clinical significance in this patient of younger age. Follow-up as clinically indicated. | 3.7 |
|  |  | PECertainty | 1. No evidence of pulmonary embolism. 2. No pneumonia. 3. Scattered left lung pulmonary nodules largest 6 mm, likely of doubtful clinical significance in this patient of younger age. Follow-up as clinically indicated. | 2.3 |
| 5 | Definitive  Negative | LLM | 1. No evidence of pulmonary embolism. 2. Hepatomegaly and secondary features of portal hypertension. Status post TIPS and paraumbilical vein embolization. Unchanged nonopacification of the hepatic veins. New nonopacification of the portal vein and TIPS. 3. Interval development of moderate to large volume ascites. | 4 |
|  |  | PECertainty | 1. No evidence of pulmonary embolism. 2. Hepatomegaly and secondary features of portal hypertension. Status post TIPS and paraumbilical vein embolization. Unchanged nonopacification of the hepatic veins. New nonopacification of the portal vein and TIPS. 3. Interval development of moderate to large volume ascites. | 3.3 |
| 2 | Definitive  Positive | LLM | Small pulmonary embolism in the left upper lobe | 3 |
|  |  | PECertainty | Small pulmonary embolism in the left upper lobe | 2.3 |
| 3 | Probable | LLM | 1. Motion degraded study limiting the sensitivity in the detection of small subsegmental pulmonary emboli. Within this limitation, no acute pulmonary embolism is identified. 2. No acute pulmonary or thoracic abnormality is seen. | 2.7 |
|  |  | PECertainty | 1. Motion degraded study limiting the sensitivity in the detection of small subsegmental pulmonary emboli. Within this limitation, no acute pulmonary embolism is identified. 2. No acute pulmonary or thoracic abnormality is seen. | 3.3 |
| 4 | Probable | LLM | Extensive respiratory motion artifact degrades evaluation of the segmental and subsegmental pulmonary arteries. Allowing for this, no evidence of central or lobar pulmonary emboli. | 3.3 |
|  |  | PECertainty | Extensive respiratory motion artifact degrades evaluation of the segmental and subsegmental pulmonary arteries. Allowing for this, no evidence of central or lobar pulmonary emboli. | 2.3 |

Note. —Average rating represents the average value of the rating from three radiologists, each in the range of 1-4. LLM represents the fine-tuned gpt-3.5-turbo-0125 with in-context learning for reasoning generation. PECertainty represents the PECertainty model with integrated gradient. The primary clues are highlighted with dark red, the secondary are in light red.

**Figure S1**


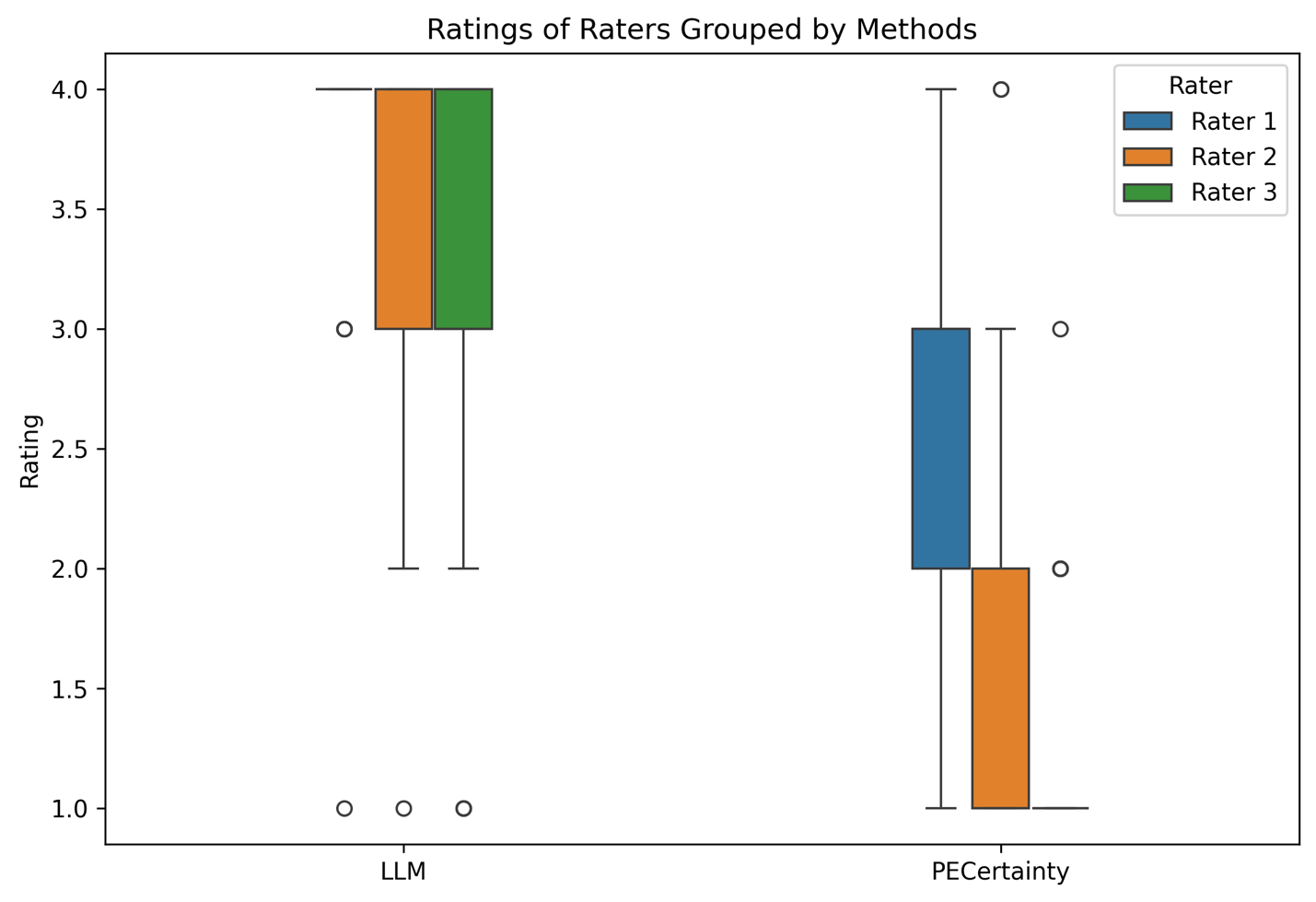


**Figure S1.** Box plot of interpretation ratings from three raters for LLM and PECertainty predictions. Each box represents the distribution of ratings for a given method, with individual raters shown in different colors. LLM predictions received consistently higher ratings across raters, while PECertainty showed more variability and lower median ratings. The boxes for Rater 1 (LLM) and Rater 3 (PECertainty) appear as single lines due to minimal variability in their ratings, which caused the box boundaries to overlap. Circles represent outliers (data points lying beyond 1.5 times the interquartile range from the first or third quartile).

**Figure S2**

**
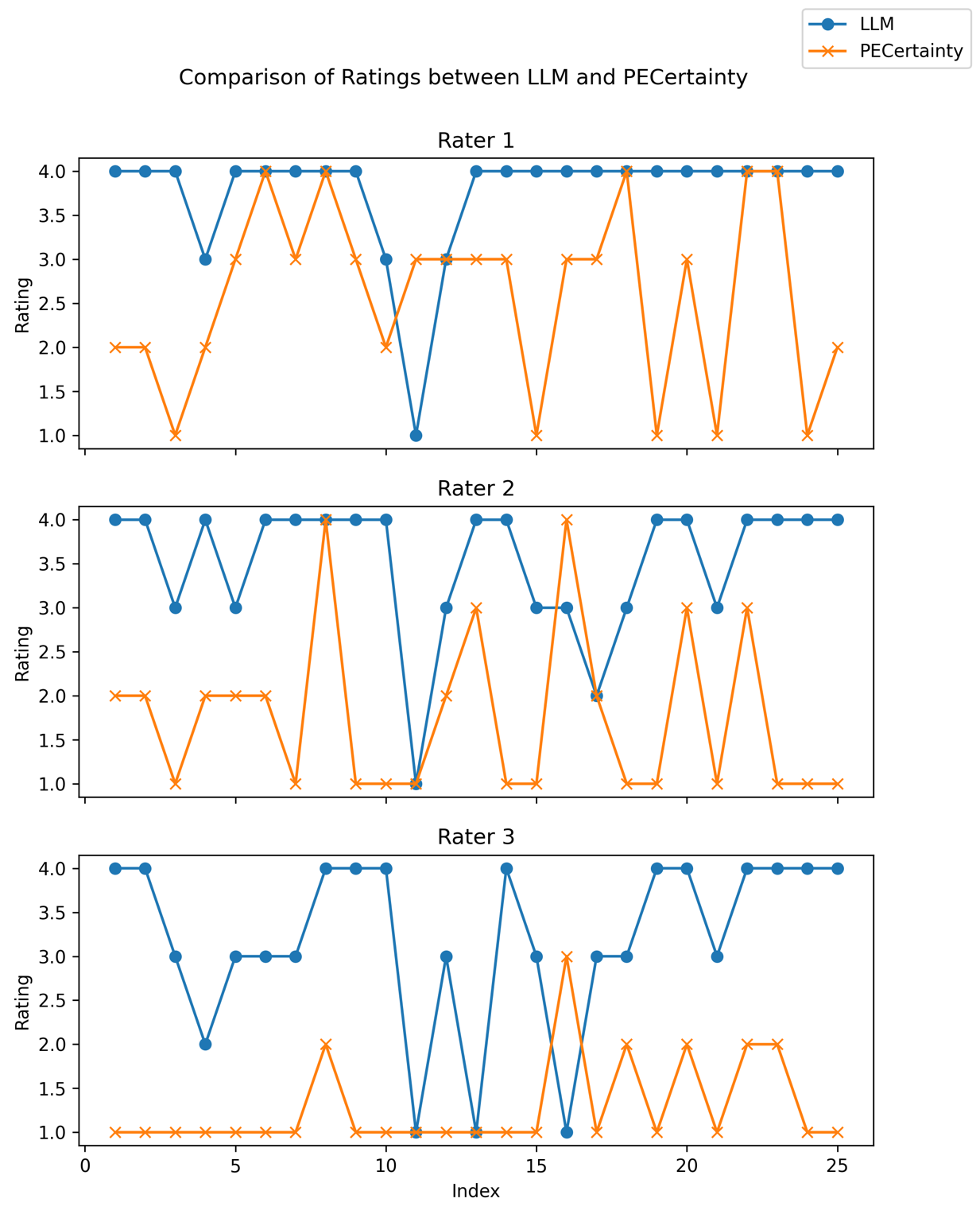
**

**Figure S2.** Line plots comparing interpretation ratings between LLM and PECertainty across each of the three raters. Each subplot displays the rating trends given by a specific rater across 25 items. LLM consistently received higher and more stable ratings across all raters, while PECertainty exhibited greater variability and lower ratings.
