# Supplementary material for: Data-Efficient Language Model for Assessing Pulmonary Embolism Diagnostic Certainty From Radiology Reports: Model Development and Validation Study
Source: JMIR Med Inform. 2026 Apr 28;14:e79972. doi: 10.2196/79972 (PMC13123884; doi:10.2196/79972)
Supplement: Multimedia Appendix 4 — Error analysis. [file medinform-v14-e79972-s004.docx]

**PECertainty** made errors in two cases, both involving misclassification of Definitive Positive reports as Definitive Negative. The fine-tuned **GPT-3.5** model misclassified three examples, predicting two Definitive Positive cases and one Definitive Negative case as Probable. Given the limited sample size, no additional consistent patterns were observed across these errors. We will continue to investigate error characteristics in future work as more data become available.

**Table S1**

| Model | Example | Prediction | Ground Truth |
| --- | --- | --- | --- |
| PECertainty | 1. Pulmonary embolism is present as described above. 2. There is no other significant abnormality. | Definitive Negative | Definitive Positive |
|  | 1. No evidence of acute pulmonary embolism. 2. Small likely chronic PE right lower lobe. No CT evidence of right heart strain. 3. Lack of opacification of left lower lobe segmental pulmonary artery branches, likely artifactual as described. 4. Left lower lobe bronchiectasis with findings suggestive of infectious bronchiolitis. | Definitive Negative | Definitive Positive |
| Fine-tuned  GPT-3.5 | 1. There is a small eccentric filling defect within a subsegmental arterial branch of the right lower lobe. The eccentric nature suggests a subacute pulmonary embolus. 2. There are subtle groundglass opacities in the dependent portions of both lungs. Findings likely represent mild alveolar edema. | Probable | Definitive Positive |
|  | Streak artifact limits evaluation. 1. No pulmonary embolism. 2. Distended main pulmonary artery suggests pulmonary hypertension. 3. Mild prominence of the interlobular septal thickening with upper lobe gradient can be attributed to interstitial edema. | Probable | Definitive Negative |
|  | 1. Linear filling defect image 217 series 5 right lower lobe and right upper lobe image 157 series 5 suggestive of either subacute or chronic pulmonary embolus. 2. Patchy right lower lobe peribronchial opacity concerning for aspiration pneumonia image 148 through 155 series 9. | Probable | Definitive Positive |
